# Loss of p120ctn causes EGFR-targeted therapy resistance and failure

**DOI:** 10.1371/journal.pone.0241299

**Published:** 2020-10-28

**Authors:** Mary E. Landmesser, Wesley M. Raup-Konsavage, Heather L. Lehman, Douglas B. Stairs

**Affiliations:** 1 Department of Pathology, The Pennsylvania State University College of Medicine, Hershey, Pennsylvania, United States of America; 2 Department of Pharmacology, The Pennsylvania State University College of Medicine, Hershey, Pennsylvania, United States of America; 3 Department of Biology, Millersville University, Millersville, Pennsylvania, United States of America; University of Central Florida College of Medicine, UNITED STATES

## Abstract

Epidermal growth factor receptor (EGFR) plays a vital role in cell division and survival signaling pathways. EGFR is activated in nearly every cancer type, and its high expression in tumors is correlated with poor patient outcome. Altogether, EGFR is a prime candidate as a therapeutic target. While targeted EGFR therapy is initially effective in 75% of patients, a majority of patients relapse within the first year due to poorly understood mechanisms of resistance. p120-catenin (p120ctn) has recently been implicated as a biomarker for EGFR therapy. In previous studies, we demonstrated that p120ctn is a tumor suppressor and its loss is capable of inducing cancer. Furthermore, p120ctn down-regulation synergizes with EGFR overexpression to cause a highly invasive cell phenotype. The purpose of this present study was to investigate whether p120ctn down-regulation induced EGFR therapeutic resistance. Using human esophageal keratinocytes, we have found that EGFR-targeting compounds are toxic to cells overexpressing EGFR. Interestingly, these therapies do not cause toxicity in cells with EGFR overexpression and decreased p120ctn expression. These data suggest that decreased p120ctn causes resistance to EGFR therapy. We believe these findings are of utmost importance, as there is an unmet need to discover mechanisms of EGFR resistance.

## Introduction

Epidermal growth factor receptor (EGFR) mediates intracellular signaling pathways that regulate cell proliferation and survival [1, 2]. Therefore, it is activated in many types of epithelial cancers, including head and neck, esophageal, lung, liver, pancreatic, colon, skin, and bladder [2, 3]. EGFR is over-activated by mutation, amplification, or overexpression, and high levels of EGFR in tumors and metastases is correlated with poorer patient outcome [2]. This makes it an ideal candidate for targeted therapies.

In 2003, gefitinib was the first EGFR-targeted compound approved by the FDA for non-small cell lung cancer (NSCLC) treatment [4]. Since then, several other small molecule EGFR inhibitors have been developed and used to treat a variety of cancers, including erlotinib, afatinib, lapatinib, and dacomitinib [5, 6]. After the introduction of small molecule EGFR-targeting compounds (Tyrosine Kinase Inhibitors (TKI)), which bind the intracellular domain of EGFR and inhibit the tyrosine kinase, monoclonal antibodies targeting the extracellular domain of EGFR were developed, including cetuximab and panitumumab. These therapies have been successful in increasing survival in patients, especially when paired with other treatments [7]. While EGFR therapies have exhibited much clinical efficacy, unfortunately the majority of patients suffer relapse or have outright treatment failure [8].

There are several factors that can prevent therapy effectiveness. The molecular mechanisms by which EGFR-targeted therapies fail can vary from mutations that occur within EGFR, such as the T790M mutation, activation of pathways that circumvent the signaling cascade such as amplification of c-MET, and the blocking of other pathways necessary for EGFR-mediated apoptosis, like the suppression of BH3 through a deletion in BIM [9–11]. While these known mechanisms of EGFR therapy resistance account for approximately 70% of patient relapses/failures, these molecular events do not explain the mechanisms of resistance in approximately 30% of patients with tumor recurrence [8]. This suggests that there must be other mechanisms of failure.

Herein, we report that p120-catenin (p120ctn) regulates EGFR therapy effectiveness. p120ctn is a tumor suppressor gene that is down-regulated or lost in a vast array of epithelial cancers, including NSCLC, head and neck squamous cell carcinoma (HNSCC), esophageal and colon cancers, and is associated with advanced stage, metastasis and poor patient prognosis [12–30]. We were the first to show that loss of p120ctn in mice leads to cancer [12]. We have previously shown that down-regulation of p120ctn in the presence of EGFR activation induces carcinogenesis and increases invasion [31]. Specifically, this combination of decreased p120ctn and increased EGFR expression induces NFkB hyperactivation synergistically, leading to increased cellular invasion [31, 32]. Highlighting the distinct relationship between p120ctn and EGFR, it was recently demonstrated that decreased p120ctn protein expression is correlated with poorer EGFR therapy survival [33].

The current data demonstrate that p120ctn is not just a marker for EGFR therapy effectiveness. Using genetically modified human esophageal squamous keratinocytes (EPC cells), the data demonstrate that down-regulation/loss of p120ctn protein expression causes resistance to EGFR therapy. Specifically, the data show that downregulation of p120ctn in cells with EGFR overexpression prevents cell death following treatment with gefitinib, erlotinib, or cetuximab.

## Materials and methods

### Cell lines

EPC1-hTERT and EPC2-hTERT cells were a gracious gift from Anil K. Rustgi (Columbia University, New York City, NY) [1, 34]. Cells were maintained at 37°C and 5% CO_2_ and were grown in keratinocyte serum-free medium (Invitrogen, Carlsbad, CA) supplemented with 40 ug/ml bovine pituitary extract, 1.0 ng/ml EGF, 100 U/ml penicillin, and 100 ug/ml streptomycin. EPC1-hTERT and EPC2-hTERT cells were genetically modified and grown as previously described [31]. Briefly, down-regulation of p120ctn in EPC1-hTERT and EPC2-hTERT cells was achieved via infection with TRIPZ inducible lentiviral p120ctn shRNA. Treatment with 1 ug/mL doxycycline for 72 hours induces the down-regulation of p120ctn in the EPC1/EPC2-P and EPC1/EPC2-PE cells. EPC1 and EPC2 cells overexpressing activated EGFR were generated by infection with the mutant EGFR-containing vector pLVX-IRES-Neo-EGFRdel, creating EPC1/EPC2-E cells [31]. EPC1-PE and EPC2-PE cells were created by using both individual techniques for p120ctn down-regulation and EGFR overexpression in the same cell. A549 lung carcinoma cells with wild type levels of EGFR were used as negative control cells for all experiments. HCC827 lung adenocarcinoma cells, with an acquired mutation in the EGFR tyrosine kinase domain, were used as a positive control for all experiments.

### Dose response curves

Cells were seeded at a density of 10,000 cells/well in a 96-well plate and allowed to attach for eight hours prior to drug treatment. Cells were treated with varying concentrations of gefitinib: 33 μM, 18 μM, 10 μM, 3.3 μM, and 1.0 μM, or BAY 11–7085: 100 μM, 33 μM, 10 μM, 3.3 μM, and 1.0 μM. DMSO vehicle controls were included. The final DMSO concentration was 1% in all conditions. Viability was measured after 48 hours using the MTS ((3-(4,5-dimethylthiazol-2-yl)-5-(3-carboxymethoxyphenyl)-2-(4-sulfophenyl)-2H-tetrazolium) assay (Biovision; Milpitas, CA) [35].

### Viability assay

Cells were seeded at a density of 10,000 cells/well in a 96-well plate and allowed to attach for eight hours prior to drug treatment. For viability testing, cells were treated with 5 μM gefitinib (#076091; Matrix Scientific, Columbia, SC), 10 μM erlotinib (#10483; Cayman Chemical; Ann Arbor, MI), or 10 nM cetuximab (#NBP2-75903; Novus Biologicals; Centennial, CO), with or without BAY 11–7085 (#B3033; ApexBio Technology; Houston, TX) at 2 μM or 3.3 μM for 48 hours. DMSO vehicle controls were included. For all treatments the final DMSO concentration was 1%. After 48 hours cell viability was assessed using the MTS assay (Biovision; Milpitas, CA) as previously described [35]. Viability was calculated by normalizing each cell line to vehicle treatment alone.

### Apoptosis assay

Cells were seeded as described previously for the viability assay. 10 uM staurosporine treatment was used as an apoptosis positive control. After 40 hours of treatment apoptosis was measured using the RealTime-Glo Annexin V kit (Promega; Madison, WI) following the manufacturer’s instructions. Luminescence and fluorescence were measured every hour from 40 to 48 hours post-treatment. Apoptosis was measured by an increase in luminescence with a delay in fluorescence, while necrosis was measured by an increase in both simultaneously.

### Western blot analysis

Cell lines were analyzed for protein expression levels of p120ctn, EGFR, phospho-NFkB, and total NFkB as previously described [31, 32] and as follows. Cells were harvested by trypsinization and centrifuged at 1,000 rpm for 5 minutes. Cell pellets were washed in 1X PBS and centrifuged at 2,500 rpm for 5 minutes. Cells were then incubated in lysis buffer and protein concentrations were determined using a Coomassie Protein Assay Kit (Pierce Biotechnology; Waltham, MA). After denatured proteins were resolved by SDS-PAGE and transferred to a polyvinylidene difluoride (PVDF) membrane, membranes were incubated overnight at 4°C with primary antibodies in a 5% BSA-PBST solution. Fluorochrome-conjugated secondary antibodies were placed on the membranes for 2 hours at room temperature in 5% BSA-PBST. Membranes were washed with 1X PBST followed by 1X PBS, and proteins were visualized using the Typhoon FLA 9000 fluorescence detection system (GE Healthcare; Chicago, IL). Antibodies against p120ctn (1:10,000; #610134; BD Transduction Laboratories, San Jose, CA), EGFR (1:5000; #4267), NFkB (1:1000; #4764), and pNFkB (1:1000; #3033) (Cell Signaling Technology, Beverly, MA) were used. β-Actin was used as a loading control (1:10,000; #A5316; Sigma Aldrich Corp., St. Louis, MO).

### Statistical analysis

For studies involving gefitinib only, the Student’s *t*-test was used to determine statistical significance and the threshold for significance was p≤0.05. For the studies in which all three EGFR inhibitors were used, an ANOVA was first performed followed by the Student’s *t*-test, if appropriate, with the same threshold as above. GraphPad Prism software (GraphPad Software; San Diego, CA) was used for statistical analysis and calculation of IC_50_ values.

## Results

### p120ctn down-regulation and EGFR overexpression in EPC1 and EPC2 cells

**Fig 1:**
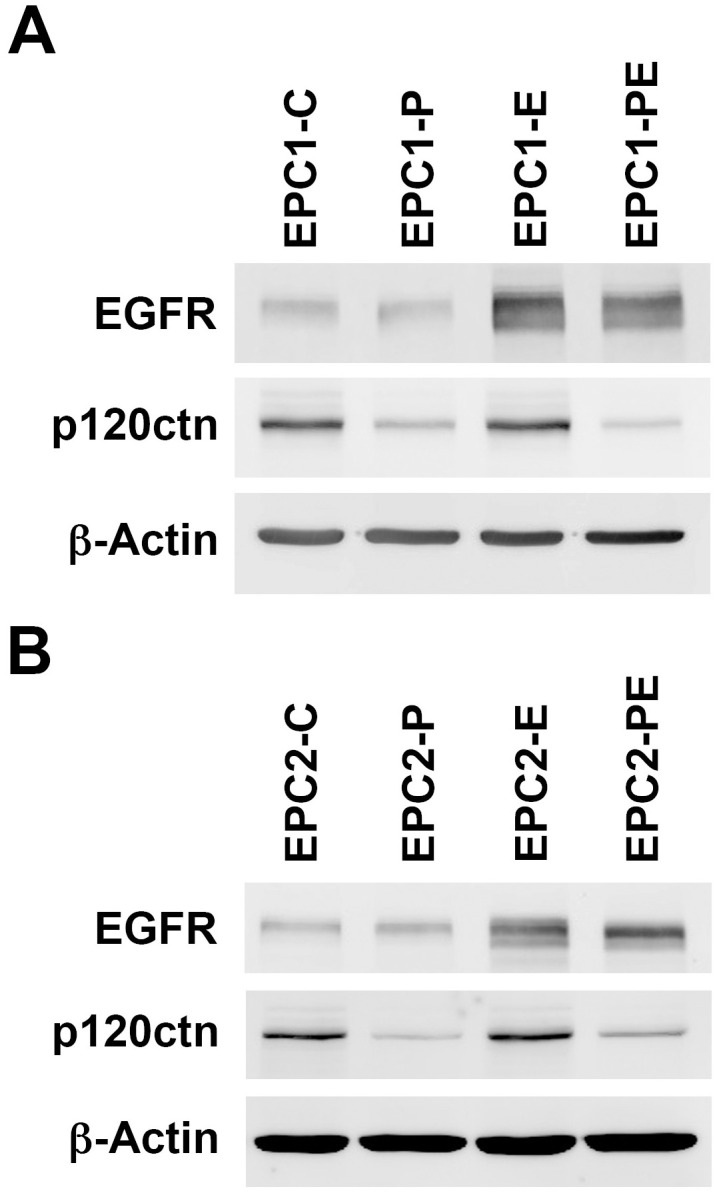
EPC cells have down-regulation of p120ctn and/or overexpression of EGFR. (A) Western blot analysis of EPC1 cells generated with modifications of p120ctn and EGFR expression confirms down-regulation of p120ctn in EPC1-P and EPC1-PE cells. EGFR is overexpressed in EPC1-E and EPC1-PE cells. (B) Western blot analysis of EPC2 cells demonstrates down-regulation of p120ctn in EPC2-P and EPC2-PE cells. EGFR overexpression is seen in EPC2-E and EPC2-PE cells. β-actin is used as a loading control.

Human esophageal keratinocyte cell lines with p120ctn down-regulation and/or EGFR overexpression were generated in both EPC1-hTERT and EPC2-hTERT parental cells, outlined in Table 1. An inducible lentiviral p120ctn shRNA was used for the knockdown of p120ctn, while EGFR overexpression was attained by transducing cells with an EGFR del mutant (E746-A750). These modifications were done individually to create knockdown of p120ctn in EPC-P cells or overexpression of EGFR in EPC-E cells, respectively. These modifications were also done in combination to create EPC-PE cells that have knockdown of p120ctn and overexpression of EGFR. The control cells (EPC1/EPC2-C) contain empty vectors and express wild type levels of p120ctn and low levels of EGFR (Fig 1). Western blot analysis demonstrates confirmation of p120ctn knockdown in EPC1/EPC2-P cells and overexpression of EGFR in EPC1/EPC2-E cells (Fig 1). EPC1/EPC2-PE cells have both down-regulation of p120ctn and overexpression of EGFR (Fig 1). In addition, Western blot analysis was used to confirm p120ctn and EGFR status prior to all experimental treatments.

**Table 1:** p120ctn and EGFR status in EPC1 and EPC2 human esophageal keratinocytes.

Cell line (EPC1/EPC2)	Genetic modification	p120ctn status	EGFR status
C	Empty vectors	wild type	wild type
P	p120ctn shRNA	knockdown	wild type
E	EGFR del (E746-A750)	wild type	overexpressed
PE	p120ctn shRNA & EGFR del (E746-A750)	knockdown	overexpressed

### Decreased p120ctn protein expression causes resistance to gefitinib

**Fig 2:**
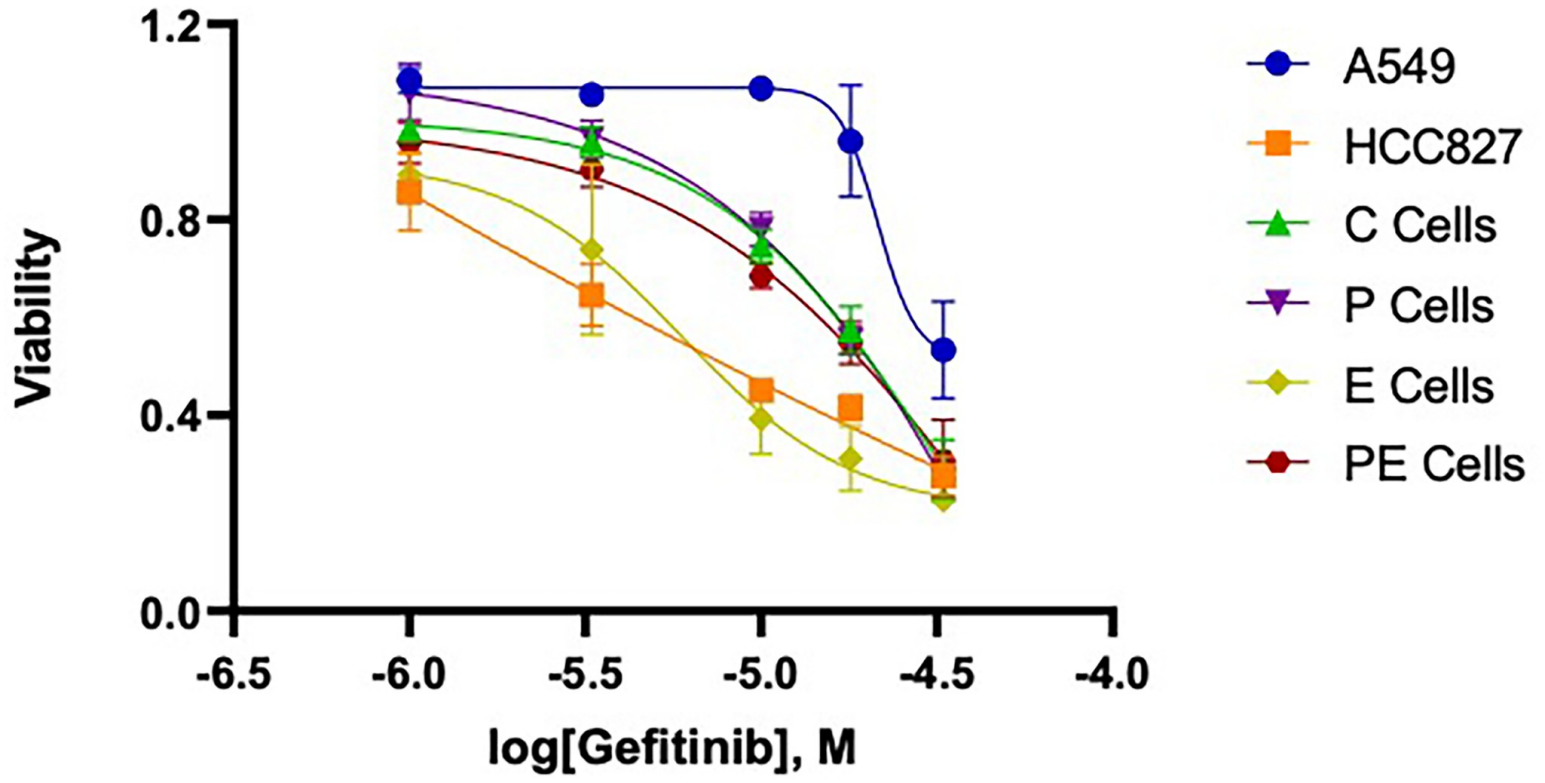
Cells with down-regulated p120ctn and EGFR overexpression are resistant to EGFR therapy-induced cell death. A dose response curve for gefitinib using EPC2 cells demonstrates that EPC2-C and -P cells with wildtype levels of EGFR were resistant to gefitinib. EPC2-E cells with activated EGFR were sensitive to treatment. EPC2-PE cells with activated EGFR and down-regulated p120ctn were resistant to EGFR therapy. Control cell lines A549 (EGFR therapy-resistant control) and HCC827 (EGFR therapy-sensitive control) responded as expected to gefitinib treatment (n = 3).

To determine whether decreased expression of p120ctn causes resistance in the engineered esophageal keratinocytes or is merely a potential biomarker for poor prognosis for patients treated with EGFR therapy as previously reported [33], we first tested the viability of EPC2-C, -P, -E, and -PE cells with varying concentrations of gefitinib to generate a dose response curve (Fig 2). Following treatment for 48 hours with gefitinib at concentrations of 1uM, 3uM, 10uM, 18uM, and 33uM, we used the MTS assay as a test of viability. A549 and HCC827 cells served as negative control and positive control cell lines for EGFR therapy, respectively. As expected, A549 cells were resistant to gefitinib and HCC827 cells were effectively killed with low doses of gefitinib, reflecting that A549 cells have wildtype EGFR and HCC827 cells have a mutant form of EGFR, as previously reported [36–38]. EPC2-C and -P cells, with wildtype EGFR expression, are also resistant to gefitinib therapy. Calculated IC_50_ values for EPC2-C and EPC2-P cells are 15 uM and 23 uM, respectively. Gefitinib was able to effectively kill EPC2-E cells overexpressing mutant EGFR with an IC_50_ value of 5.8 uM. Interestingly, EPC2-PE cells, with the combination of decreased p120ctn expression and EGFR overexpression, were resistant to gefitinib treatment. The IC_50_ value of EPC2-PE cells was 24.6 uM. In fact, EPC2-PE cells have a toxicity profile that is indistinguishable from control EPC2-C and EPC2-P cells, suggesting a strong resistance to gefitinib induced by decreased p120ctn expression. These data establish that decreased p120ctn expression in human esophageal keratinocytes can cause gefitinib resistance.

### Decreased p120ctn-induced EGFR resistance affects both EGFR-TKI and EGFR monoclonal antibody therapy

**Fig 3:**
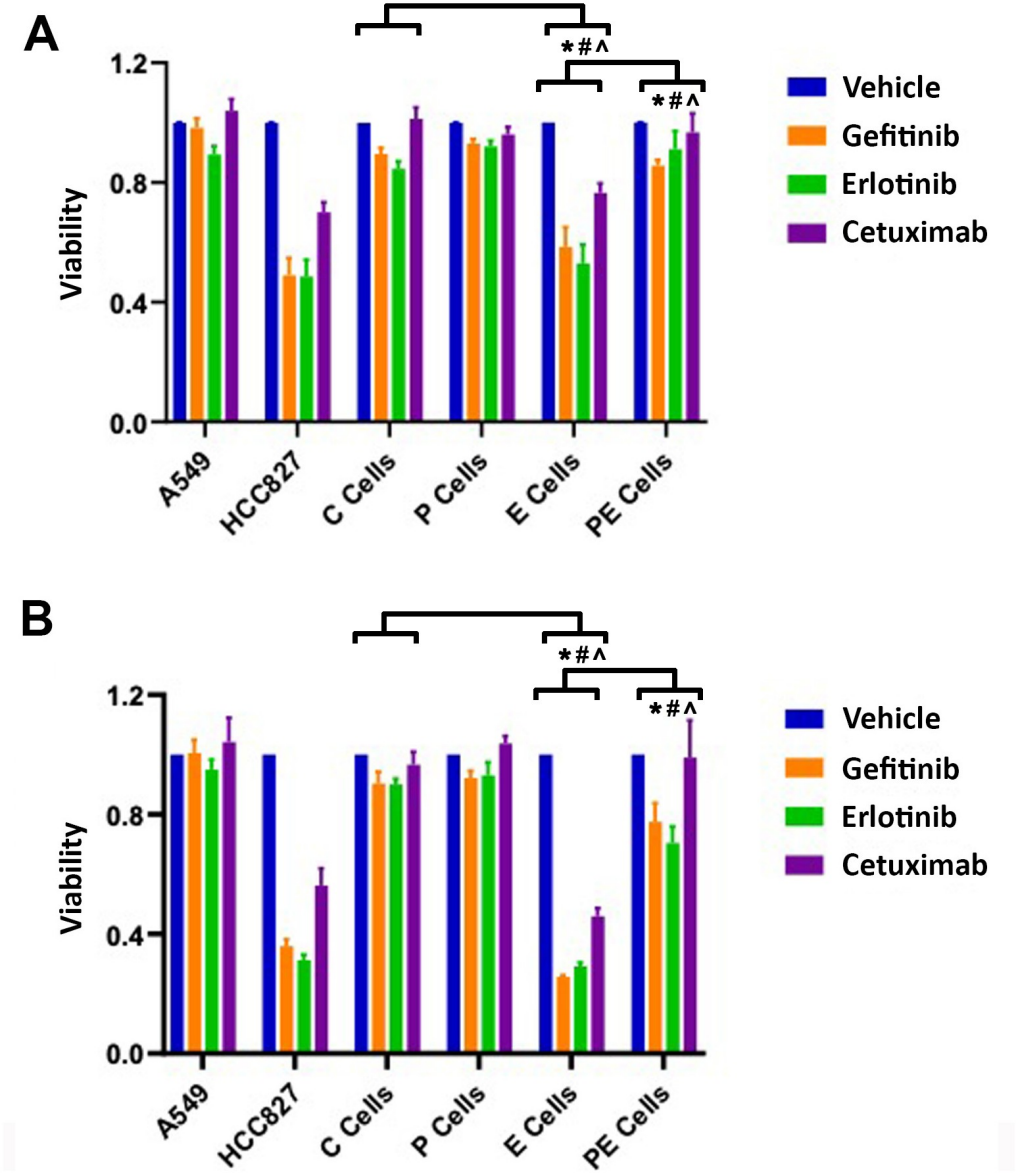
EPC cells with both down-regulated p120ctn and EGFR overexpression are resistant to cell death when treated with gefitinib, erlotinib, or cetuximab. (A) Cell viability assays in EPC1-C, -P, -E, and -PE cells show that EPC1-PE cells are resistant to EGFR therapy-induced death, (n = 8). (B) Cell viability assays show that EPC2-PE cells are resistant to cell death, (n = 4). Vehicle control was DMSO. * denotes p<0.05 for gefitinib treatment comparisons between EPC-E vs. EPC-C and EPC-PE vs. EPC-E. # denotes p<0.05 for erlotinib treatment comparisons between EPC-E vs. EPC-C and EPC-PE vs. EPC-E. ^ denotes p<0.05 for cetuximab treatment comparisons between EPC-E vs. EPC-C and EPC-PE vs. EPC-E.

Small molecule EGFR inhibitors like gefitinib are tyrosine kinase inhibitors, working through inhibition of the activity of tyrosine kinase on the intracellular domain of the EGFR. Monoclonal antibodies to EGFR bind the extracellular portion of EGFR to inhibit overexpression. After establishing that PE cells exhibited resistance to gefitinib, we assessed other EGFR-targeted therapies in the engineered cell lines to determine the effect of combining down-regulated p120ctn with overexpressed EGFR on cell viability. In addition to gefitinib, we treated EPC1 and EPC2 cells with the TKI erlotinib or the EGFR monoclonal antibody cetuximab. Cells were treated with each inhibitor at the following concentrations: gefitinib 5uM, erlotinib 10uM, cetuximab 10nM, or DMSO vehicle alone. Control cell lines performed as anticipated; A549 cells exhibited resistance to gefitinib, erlotinib, and cetuximab while the HCC827 cells were susceptible to the treatments (Fig 3). As with gefitinib, the EPC1-C and -P cells expressing wildtype EGFR exhibited resistance to the other EGFR inhibitors, erlotinib and cetuximab. The EPC1-E cells overexpressing EGFR had inhibited growth with all EGFR therapies. Importantly, EPC1-PE cells, expressing down-regulated p120ctn and upregulated EGFR, demonstrated resistance to all EGFR inhibitors tested, and that resistance was not statistically different than that found in EPC1-C and -P cells (Fig 3A). This experiment was repeated and validated using an independent EPC2 cell line, and we observed similar results (Fig 3B). These data suggest that p120ctn confers resistance to not just gefitinib, but to multiple TKIs and other classes of EGFR-targeted therapies.

**Fig 4:**
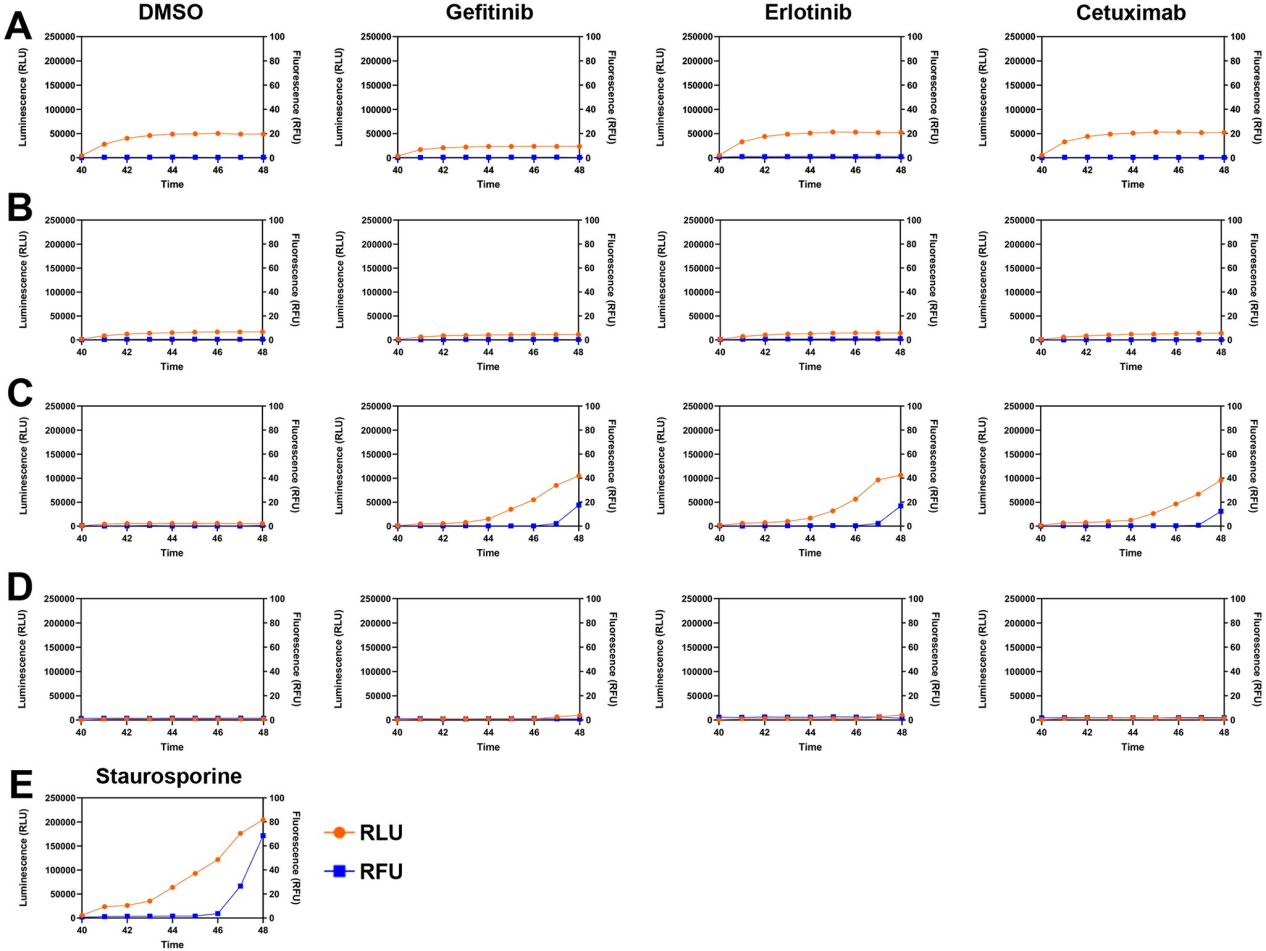
EPC1-E cells overexpressing EGFR undergo apoptosis when treated with gefitinib, erlotinib, or cetuximab. (A) Annexin V assays measured over 48 hours show a slight increase in luminescence in EPC1-C cells. (B) EPC1-P cells demonstrate resistance to cell death. (C) Annexin V apoptosis assays show that EPC1-E cells undergo apoptosis when treated with EGFR inhibitors. (D) EPC1-PE cells are resistant to apoptosis, as measured by Annexin V. (E) Staurosporine was used as a positive control. For all apoptosis experiments, n = 3.

Since the EPC1-E cells overexpressing EGFR had decreased viability with all EGFR therapy treatments, we sought to determine whether the cells were undergoing apoptosis or necrosis. After performing an Annexin V apoptosis assay, we showed that compared to EPC1-C and EPC1-P cells, EPC1-E cells were indeed undergoing apoptosis as a result of treatment with all three EGFR inhibitors (Fig 4A–4C). EPC1-E cells showed an increase in luminescence prior to fluorescence, similar to the Staurosporine positive control (Fig 4E). Results of this assay confirmed that EPC1-PE cells that have down-regulated p120ctn and overexpressed EGFR are resistant to cell death when treated with EGFR inhibitors (Fig 4D).

### NFkB signaling may mediate EGFR therapy resistance induced by decreased p120ctn expression

**Fig 5:**
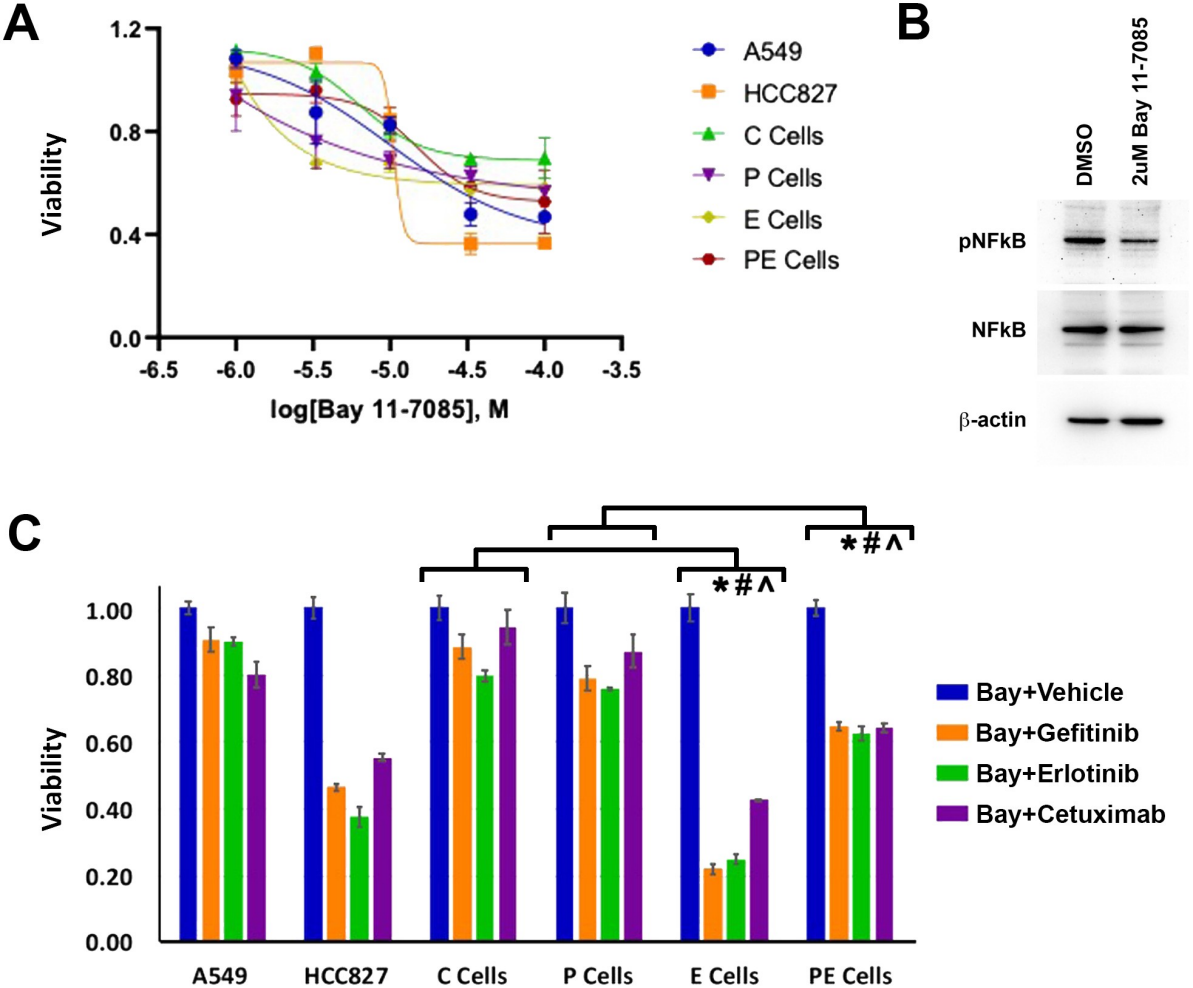
Combined inhibition of EGFR and NFkB induces partial lethality in cells with down-regulated p120ctn and overexpressed EGFR. (A) A dose response curve for BAY 11–7085 using EPC1 cells demonstrates no significant cell death in any cell line at 2 uM. (B) Western blot analysis demonstrates a decrease in pNFkB expression when cells are treated with 2 uM BAY 11–7085. (C) Cell viability assays demonstrate that treatment of EPC1 cells with 2 uM BAY 11–7085 in combination with either gefitinib, erlotinib, or cetuximab results in a partial reduction in cell viability in EPC1-PE cells (n = 3). Vehicle refers to cells treated with 2 uM BAY 11–7085 and DMSO (as the EGFR diluent). * denotes p<0.05 for gefitinib treatment comparisons between EPC-E vs. EPC-C and EPC-PE vs. EPC-E. # denotes p<0.05 for erlotinib treatment comparisons between EPC-E vs. EPC-C and EPC-PE vs. EPC-E. ^ denotes p<0.05 for cetuximab treatment comparisons between EPC-E vs. EPC-C and EPC-PE vs. EPC-E.

Having previously established that NFkB is hyperactivated by the cooperation between p120ctn down-regulation and EGFR overexpression [32], along with the known pro-survival effects of NFkB, we hypothesized that the combination of NFkB and EGFR inhibition may be able to induce EPC1-PE cell death. BAY 11–7085 is an NFkB inhibitor that irreversibly inhibits activation by blocking phosphorylation of IkB-α [39, 40]. We began by generating a dose response curve with BAY 11–7085 to determine a concentration that would not be lethal to the cells on its own. Treating the cells with the following concentrations of BAY 11–7085: 1 uM, 3.3 uM, 10 uM, 33 uM, and 100 uM, we generated a growth curve as determined by MTS assay (Fig 5A). The BAY 11–7085 IC_50_ values for EPC1-C, -P, and -PE cells were 6.2 uM, 5.9 uM, and 15.1 uM, respectively. The IC_50_ value for EPC1-E cells was >100uM, as the highest value we tested did not kill 50% of the cells. Based on these values, we chose to co-treat the control and EPC1 cell lines with 2 uM BAY 11–7085 and the EGFR therapies as previously described. On average, treatment with BAY 11–7085 inhibited NFkB activity by 3.2 fold, as demonstrated by Western blot analysis in Fig 5B. The addition of BAY 11–7085 with EGFR inhibitors had a minor but not statistically significant effect on the viability of EPC1-C or EPC1-P cells—approximately 0.8 with all three EGFR inhibitors versus with BAY 11–7085 and vehicle (DMSO as an EGFR inhibitor diluent) (Fig 5C). The addition of BAY 11–7085 with EGFR inhibitors induced a significant amount of cell death in EPC1-E cells, 0.3–0.4 viability (Fig 5C) versus BAY 11–7085 and vehicle. Interestingly, the EPC1-PE cells, expressing low levels of p120ctn and high levels of EGFR, had an intermediate viability when treated with both BAY 11–7085 and EGFR inhibitors (Fig 5C). The viability of EPC1-PE cells treated with BAY 11–7085 and EGFR inhibitors is significantly reduced, approximately 0.6, when compared to the BAY 11–7085 and vehicle control, 1.0, and to EPC1-C and EPC1-P cells, approximately 0.8 (Fig 5C). While viability is reduced in EPC1-PE cells treated with BAY 11–7085 and EGFR inhibitors, they are not as severely affected as EPC1-E cells. These data suggest that either NFkB signaling is not solely responsible for EGFR therapy resistance induced by p120ctn or we have not achieved an optimal therapeutic dose. Unfortunately, treatment with higher BAY 11–7085 concentrations is prohibitive, this and other NFkB inhibitors have been well documented to cause problems in clinical trials by inducing unintended cytotoxicities and immune suppression [32, 41, 42].

## Discussion

Given its role in diverse cellular processes and its activation in a wide array of cancers, EGFR stands out as a prime therapeutic target. Small molecule tyrosine kinase inhibitors (such as gefitinib and erlotinib) and monoclonal antibodies (such as cetuximab), each with their distinct mechanisms of action, have been developed for use in targeting EGFR in a number of cancers [43, 44]. While each of these EGFR-targeted therapies have demonstrated clinical efficacy in a number of contexts, including an increase in overall survival and progression-free survival, therapy failure still occurs [8, 45–48]. Tumors frequently develop acquired resistance due to secondary mutations in the targeted gene or other mutations contained within the tumor cell [9, 10, 49]. There is a crucial need to identify and understand these resistance mechanisms and identify the genes and mutations that can modify the effectiveness of EGFR therapy.

Our previous work identified p120ctn as a tumor suppressor and that its down-regulation/loss induces cancer *in vivo* [12]. Moreover, we demonstrated that p120ctn down-regulation intersects and synergizes with EGFR overexpression to induce an aggressive and invasive cancer phenotype [31]. Even more recently it has been indicated that p120ctn may be a biomarker for EGFR therapy in NSCLC. Specifically, lung cancer patients with low p120ctn protein expression had poorer outcomes when treated with gefitinib than those with normal p120ctn expression [33]. Given the transcriptional and carcinogenic interaction between p120ctn and EGFR, we hypothesized that p120ctn was more than a biomarker of EGFR therapy; rather, p120ctn could induce EGFR therapy resistance. In the present study, we show that the EGFR-targeted therapies, gefitinib, erlotinib, and cetuximab, are toxic to cells overexpressing EGFR alone but are not toxic at the same dose to cells with EGFR overexpression combined with p120ctn down-regulation. These data demonstrate that decreased p120ctn causes resistance to EGFR therapy.

The molecular underpinnings of the cooperation between p120ctn and EGFR may play a role in the ability of p120ctn to affect EGFR therapy efficacy. We previously showed that down-regulation of p120ctn combined with overexpression of EGFR induces hyperactivation of NFkB p65 in human esophageal keratinocytes [32]. NFkB is a transcription factor that is classically known to regulate genes involved in normal cellular processes and oncogenic processes, including proliferation, invasion, survival and apoptosis [50, 51]. With this in mind, we hypothesized that a combination therapy of EGFR inhibitors and an NFkB inhibitor may induce cell death in cells with down-regulated p120ctn and overexpressing EGFR that were otherwise resistant to EGFR therapy. The initial data show that combined NFkB inhibitor with the EGFR therapies lead to an intermediate amount of cell death in cells with low p120ctn protein expression and EGFR overexpression, suggesting that an NFkB inhibitor may be useful in combination with an EGFR-targeted therapy to treat EGFR-positive tumors with decreased p120ctn. Further studies need to be conducted in order to confirm if NFkB inhibition will aid EGFR drugs in effectively killing cells with p120ctn down-regulation and EGFR overexpression.

The ability to individualize cancer therapies in an effort to increase a patient’s chance of survival is dependent upon understanding the genes and proteins that may alter therapies and affect their efficacy. Decreased p120ctn has been identified as such a biomarker, and we have shown here that decreased p120ctn can indeed cause a tumor to be resistant to EGFR-targeted therapies. Though further studies are required, these data suggest that stratifying patients with EGFR-activated tumors based on their p120ctn expression may aid in therapy effectiveness. Indeed, since decreased p120ctn protein expression significantly overlaps with EGFR overexpression in ESCC [31], it may be an explanation why cetuximab clinical trials failed in treating patients with ESCC [52]. Furthermore, inhibition of NFkB may be able to cooperate with EGFR therapy to eradicate tumors with EGFR activation and p120ctn down-regulation. Combination therapy is often the best way to increase treatment efficacy, prevent drug resistance, and reduce treatment duration for patients.

## Supporting information

S1 Raw images (PDF)
