# Supplementary material for: Loss of p120ctn causes EGFR-targeted therapy resistance and failure
Source: PLoS One. 2020 Oct 28;15(10):e0241299. doi: 10.1371/journal.pone.0241299 (PMC7592761; doi:10.1371/journal.pone.0241299)
Supplement: S1 Raw images — (PDF) [file pone.0241299.s001.pdf]

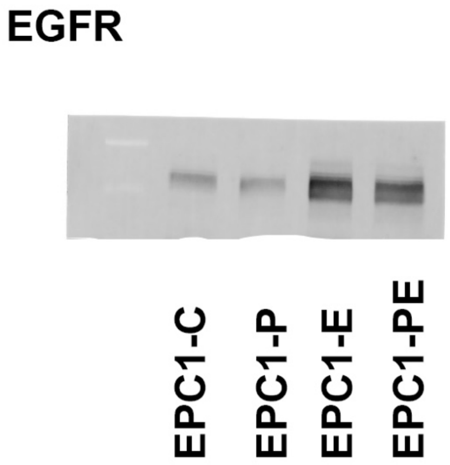

S1 Fig. EGFR in EPC1 cells. Full-length Western blot of EGFR expression in EPC1-C, -P, -E, and -PE cells, visualized by fluorescence using the Typhoon FLA 9000 system. Lanes have been labeled to match the main text.

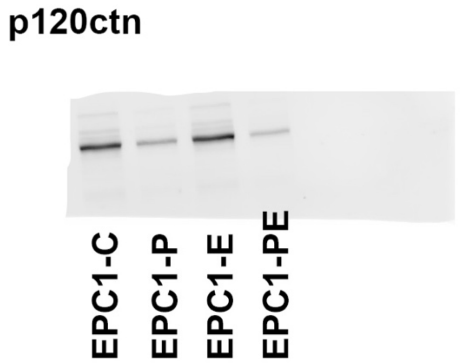

S2 Fig. p120ctn in EPC1 cells. Full-length Western blot of p120ctn expression in EPC1-C, -P, -E, and -PE cells, visualized by fluorescence using the Typhoon FLA 9000 system. Lanes have been labeled to match the main text.

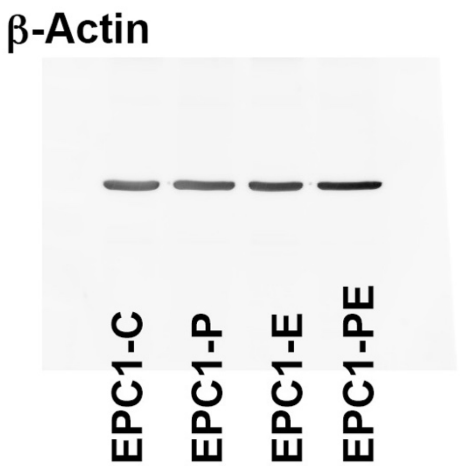

S3 Fig. β-Actin in EPC1 cells. Full-length Western blot of β-Actin expression in EPC1-C, -P, -E, and -PE cells, visualized by fluorescence using the Typhoon FLA 9000 system. β-Actin was used as a loading control for EGFR and p120ctn in EPC1 cells. Lanes have been labeled to match the main text.

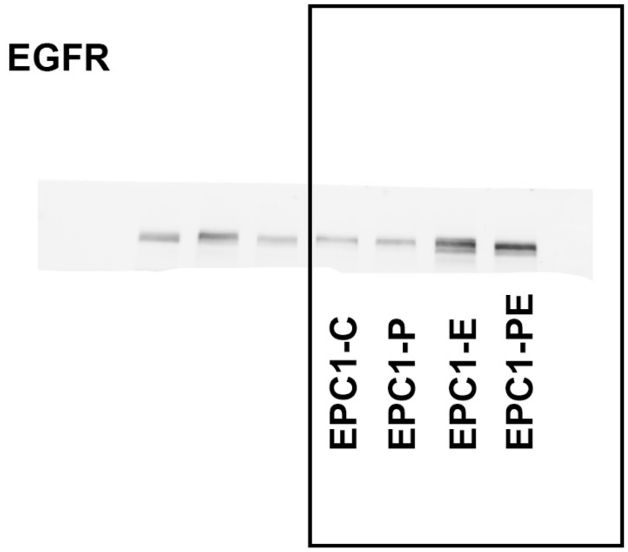

S4 Fig. EGFR in EPC2 cells. Full-length Western blot of EGFR expression in EPC2-C, -P, -E, and -PE cells, visualized by fluorescence using the Typhoon FLA 9000 system. Relevant experiment is marked with a black box and lanes have been labeled to match the main text.

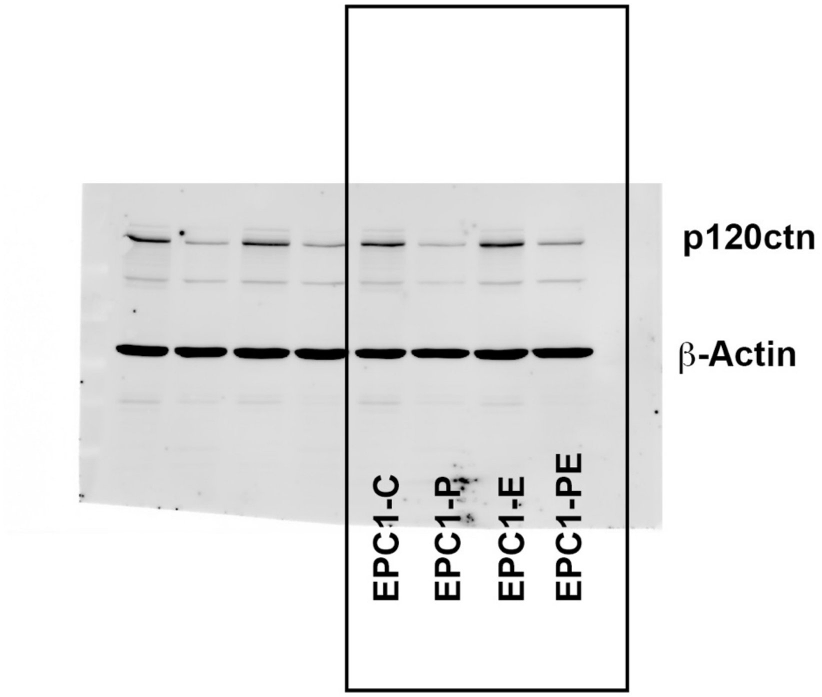

S5 Fig. p120ctn and β-Actin in EPC2 cells. Full-length Western blot of p120ctn and β-Actin expression in EPC2-C, -P, -E, and -PE cells, visualized by fluorescence using the Typhoon FLA 9000 system. β-Actin was used as a loading control for EGFR and p120ctn in EPC2 cells. Relevant experiment is marked with a black box and lanes have been labeled to match the main text.

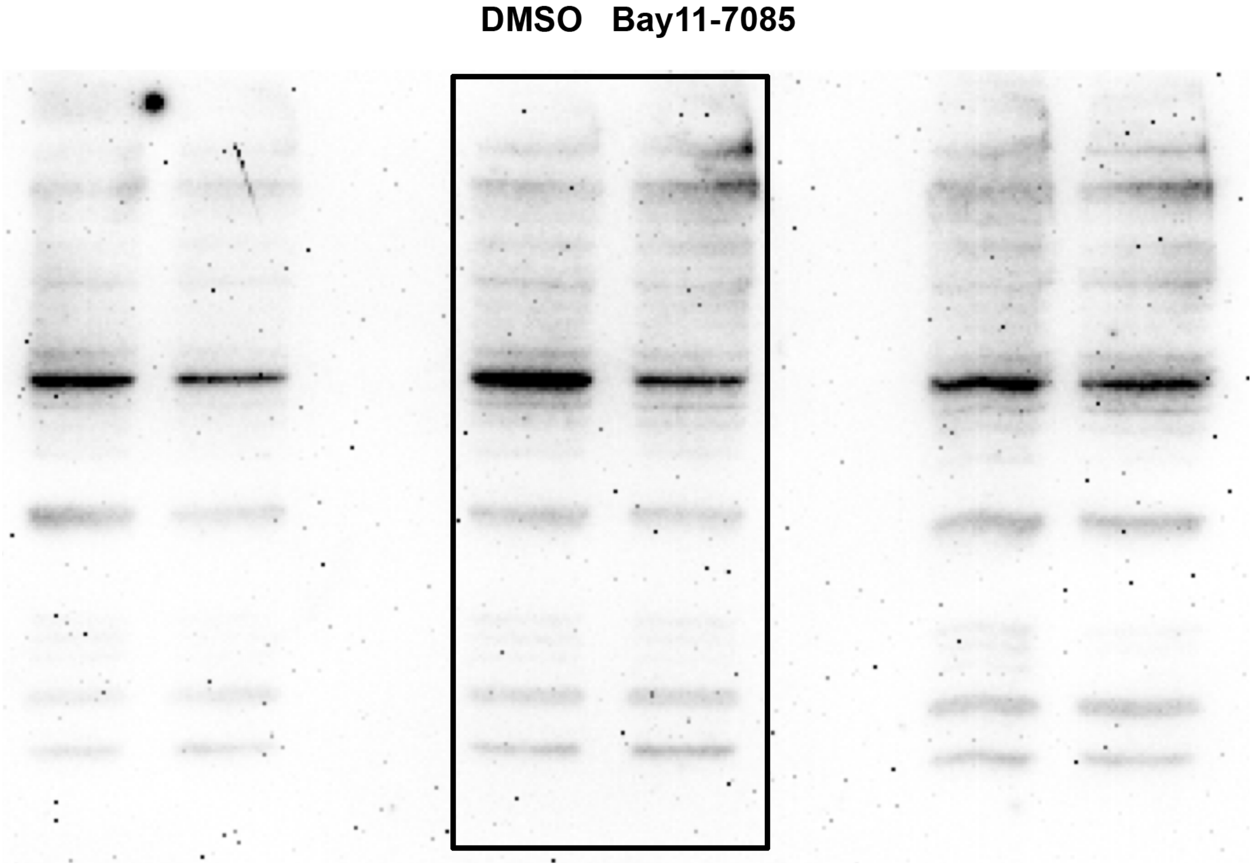

S6 Fig. pNFkB inhibition in EPC1 cells after BAY 11-7085 treatment. Full-length Western blot of pNFkB, after DMSO or 2 uM BAY 11-7085 treatment in EPC-PE cells. Relevant experiment is marked with a black box and lanes have been labeled to match the main text.

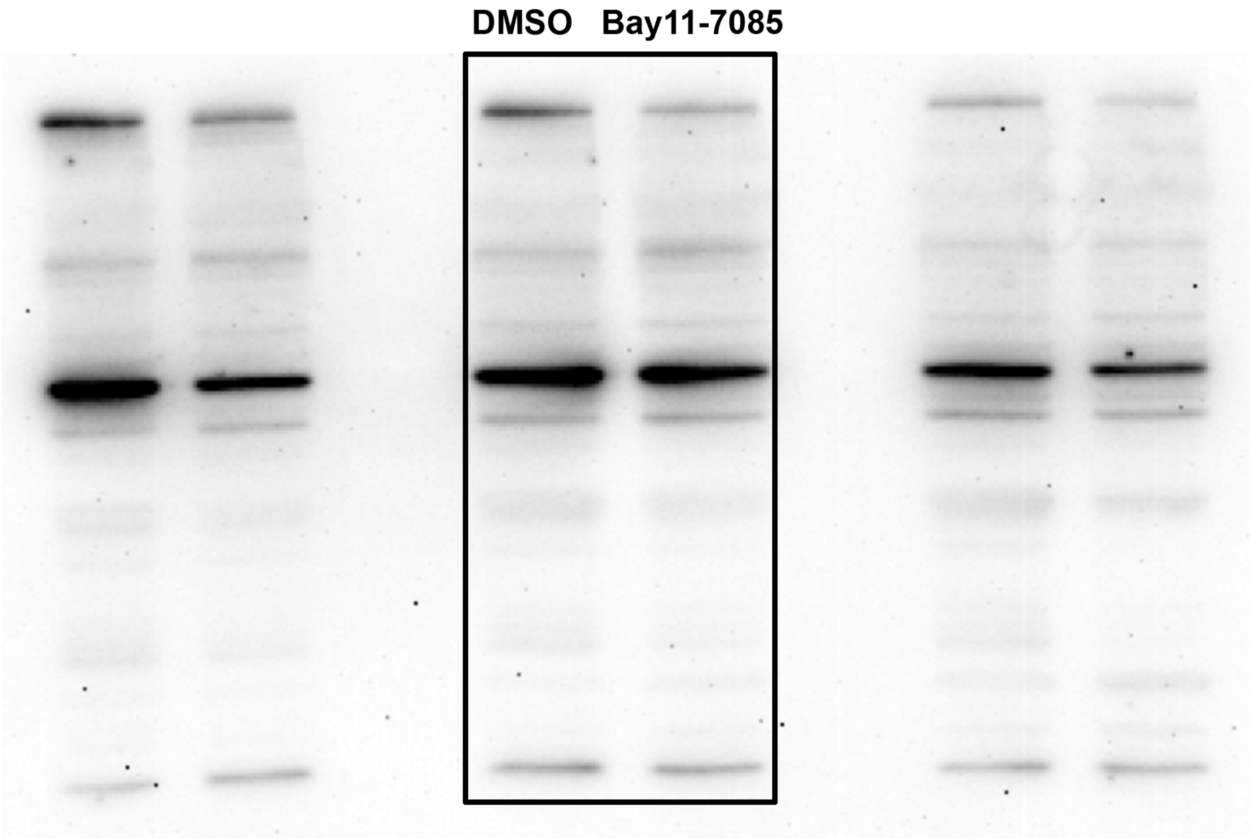

S7 Fig. pNFkB inhibition in EPC1 cells after BAY 11-7085 treatment. Full-length Western blot of NFkB after DMSO or 2 uM BAY 11-7085 treatment in EPC-PE cells. Relevant experiment is marked with a black box and lanes have been labeled to match the main text.

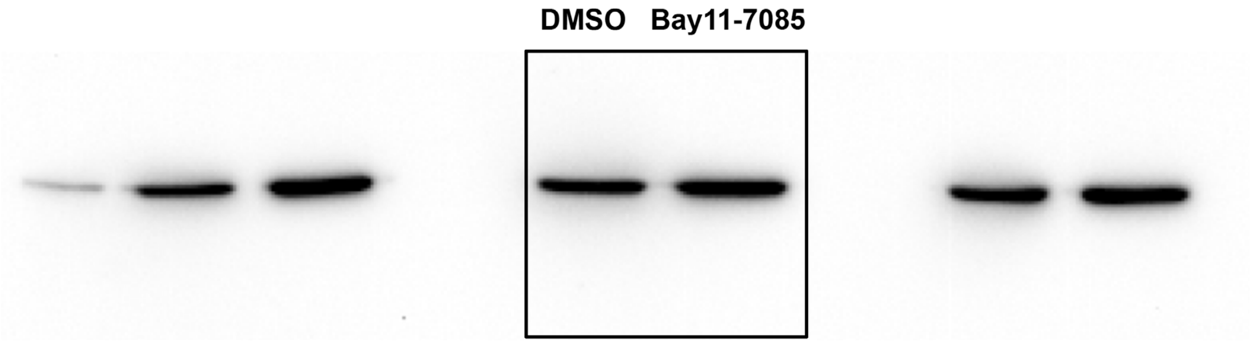

S8 Fig. pNFkB inhibition in EPC1 cells after BAY 11-7085 treatment. Full-length Western blot of β-Actin after DMSO or 2 uM BAY 11-7085 treatment in EPC-PE cells. Relevant experiment is marked with a black box and lanes have been labeled to match the main text.
